# Digestion of Albumen and Flesh, and the Comparative Anatomy and Physiology of the Pancreas

**Published:** 1856-05

**Authors:** Joseph Jones

**Affiliations:** Georgia


### MEDICAL EXAMINER.

NEW SERIES.—NO. CXXXVII.—MAY, 1856.

### ORIGINAL COMMUNICATIONS.

*Digestion of Albumen and Flesh, and the comparative Anatomy and Physiology of the Pancreas.* By JOSEPH JONES, M. D., of Georgia.

The process of digestion has been the subject of numerous careful and laborious investigations, since Spallanzani first demonstrated it to be a chemico-physical process, and after the researches of Spallanzani, Magendie, Tiedeman, Gmelin, Prout, Beaumont, Mulder, Dumas, Liebig, Blondot, Bernard, Lehmann, Bidder and Schmidt, and many others, it seems impossible that it should still remain in obscurity. It has been the unanimous opinion of all physiologists, that flesh and the protein bodies generally were digested entirely in the stomach. Recently, however, this fact has been denied by physiologists of the highest authority. By recent experiments, Bidder and Schmidt have convinced themselves that one of the important offices of the intestinal juice is to dissolve and render fit for absorption, not only starch, but also flesh and other protein bodies. They assert that the intestinal juice not only metamorphoses starch with as great rapidity as saliva and pancreatic juice, but also, that the intestine exerts as powerful a digestive influence on flesh and albumen as the stomach. Frerichs, on the other hand, has been unable in his experiments to detect any change exerted by the intestinal juice upon the protein elements of the food.

Protein bodies, gelatinous substances, fat and starch, remained unchanged, and he denies positively that the intestinal juice has any action as a direct digestive agent. Professor Lehmann, in a series of experiments upon the intestinal fluid collected from a loop of a gut from a human being, with a fistulous opening into the small intestine, found that it possessed, in a high degree, the power of converting starch into sugar, whilst protein bodies and fats were not affected in any appreciable manner. Professor Lehmann, however, attaches little importance to these experiments performed by himself, from the fact that the fistulous opening was in the lower portion of the ileum, and probably near the cæcum. He adopts the experiments of Bidder and Schmidt, and by an argument drawn from the amount of gastric juice secreted in a given length of time, and the amount of protein substances which it is capable of digesting, concludes that a large portion of flesh and albumen and the other protein bodies pass out of the stomach undigestsd, and are finally dissolved by the intestinal juice.

According to Professor Lehmann, the amount of gastric fluid secreted by a dog in 24 hours, equals one-tenth the weight of the whole body. 100 grs. of recent gastric juice are capable of dissolving from three to five grains of coagulated albumen. A dog needs daily, for the perfect maintenance of all the physiological functions, 50 grains of flesh (containing 10 grains of albuminates) for every 1,000 grains of its weight. It secretes, however, only 100 grains of gastric juice for every 1,000 grs. of its weight, only one half the amount capable of dissolving the albuminates of the flesh. Hence, a large portion of the protein bodies must pass out of the stomach undigested.

Careful experiments have shown that the gastric juice is deprived, in the duodenum, of its free acid, and with it of its power of digestion, by the bile and pancreatic fluid. Hence, other fluids must flow into the intestines which are capable of dissolving the protein bodies.

The only method of deciding accurately upon the truth of these conclusions, drawn by Prof. Lehmann, from the preceding argument, is to appeal to the physico-chemical process of digestion, as it is performed in a normal condition in the animal economy.

I have enjoyed numerous opportunities of examining the contents of the stomachs of fishes, reptiles, birds and mammalia, in every stage of the digestive process, and never have I discovered undigested particles of flesh in the small intestines. The following observations were made during the prosecution of various researches upon the blood, urine, relative weights of the organs, and comparative anatomy, and minute anatomical structure of the organs of different animals, without any reference whatever to the maintenance of a theory.

The stomach of an alligator (Alligator mississippiensis) contained the bones, teeth, hoofs and hair of a pig. The meat had been entirely digested, leaving the bones as clean as those of a prepared skeleton.

In the stomach of other alligators we have found fishes, snakes, crabs, &c., in different stages of digestion, some but slightly acted upon by the gastric juice, others partially dissolved, whilst of others little more than their bones remained.

The stomach of a bull-frog, (Rana pipiens,) which had been captured 24 hours, contained several craw fish (Astacus Bartoni) and a long slender grass snake (Tropidonotus ordinatus) about three feet in length. Although this food had been swallowed for more than 24 hours, only the exterior parts of the body of the serpent showed the evidences of the action of the gastric juice, and the shell of the invertebrate animals was of a red color, resembling that which they assume after they have been acted upon by boiling water.

In the stomachs of serpents we have found smaller serpents, lizards and mice, in all stages of digestion, whilst the small intestines contained not a particle of meat. These observations were also verified by an examination of the contents of the stomachs of fishes, carnivorous birds, as the buzzards, hawks, cranes, herons, &c., and also of carnivorous mammalia, as raccoons and dogs.

If one-half of all the meat received into the stomach passes into the small intestines, and if the process of digestion, is, according to the statement of Bidder and Schmidt, as slow as that of the stomach, why is it that its presence always eluded observation when the intestinal canal was laid open? In the case of animals which swallow their prey whole, without any mastication, how could portions of meat with large bones attached pass out of the capacious stomach into the contracted intestine, without being evident to the observer. All the observations thus far made convince me that meat is entirely digested in the stomach.

Another fact worthy of note is, that in the stomach of all animals, whether cold or warm-blooded, which I have examined during the process of digestion, the amount of fluid, containing the digested matters in solution, was exceedingly small, ofttimes amounting to only a few drops, and in many cases, especially amongst cold-blooded animals, it appeared to be almost entirely absent. This proves that, in the normal process of digestion, the matters dissolved by the gastric juice are almost immediately absorbed or pass into the duodenum.

The idea that a solution of the albuminous matters accumulates in the stomach which has been called chyme by writers, is erroneous. This fact shows the fallacy of Prof. Lehmann's argument. He calculated the amount of flesh which could be digested by a given quantity of gastric juice out of the body. In nature the process is far different from that of artificial digestion. A portion of gastric juice dissolves a definite amount of flesh, and the solution is then absorbed, or passes out into the small intestines. Another portion of gastric juice is secreted and acts upon the fresh exposed surface of the flesh, and the products of its action are in turn absorbed.

It is evident to every candid observer, that this process is far more energetic than that of artificial digestion, and consequently the one cannot be the measure of the other, and the argument founded upon artificial digestion falls to the ground.

Even granting that artificial and normal digestion are precisely similar as far as the rapidity of their actions is concerned, how is it possible for Prof. Lehmann to determine the amount of gastric juice secreted by the stomach in a given time, when absorption is almost as rapid as secretion. In the consideration of the digestion of protein bodies, he leaves this fact entirely out of view. The absorption and passage of the digested matters out of the stomach immediately after their solution, is true also of granivorous and frugivorous animals. I have examined the stomach of numerous squirrels, rats and birds, fed upon grain, acorns, nuts and berries, and in every instance there was no fluid which corresponded to the chyme of writers. Even in those animals which subsist upon grasses, and green buds, and leaves, which contain a larger amount of fluid, the contents of the stomach are comparatively dry. Almost every one has it in his power to verify the truth of these observations, by simply walking to the butcher pens and examining the contents of the stomachs of cows and sheep.

Amongst cold-blooded animals, the only frugivorous animals which I was able to examine, was the gopher, (Testudo polyphemus.) In this animal the grasses and vegetable matters appear to be principally digested in the colon.

The colon of the gopher enlarges into a receptacle for food, 30 inches in length and four inches in circumference. In one instance, after 30 days' starvation, the undigested vegetable contents of the colon and cæcum amounted to 1460 grains. The gopher has the power of retaining in its intestines vegetable food without either digesting it, or allowing it to putrefy or ferment. When, however, it is removed from the intestinal canal, in the course of a few days it putrefies and becomes filled with numerous worms. This is a provision of the Creator, adapting this animal to the habitation of a sandy and barren country, where it is often impossible to obtain water, and where vegetation is scarce. The colon contains a store of vegetable food, which replaces the wastes of the blood and tissues during long seasons of drought and starvation, and supplies the place of the masses of fat found in the abdominal cavities of many Chelonians, Ophidians and Saurians, which are consumed during long fasting.

### *Comparative Anatomy and Physiology of the Pancreas.*

In the invertebrate kingdom no lymphatic system has been discovered, and the existence of a pancreatic gland has not as yet been satisfactorily demonstrated. Siebold considers two thick-walled cæca, lined with ciliated epithelium and opening into the beginning of the stomach, in many Rotatoria, a rudimentary pancreas. Hunter, Grant, Owen, Siebold and Rhymer Jones, consider the pale yellow, ramified tubes, which in many species of Cephalapoda are appended to the hepatic ducts, as true representatives of the pancreatic glands of higher animals.

These, however, have not been certainly demonstrated to be pancreatic g'ands, for no comparative anatomist or physiologist has as yet described the special character and offices of their secretion. A rapid review of the character of the digestive apparatus and circulatory system of these animals, will show why the lymphatic system and pancreas should be absent.

In the lowest forms of the Protozoa, which are simple cells provided with vibratile ciliæ resembling closely the sporules of vegetables, we find neither organs nor a circulatory system. The highest members of this group have contractile pulsatory cavities situated in the denser and outer layers of the parenchyma of the body. No blood vessels communicate with these cavities, and no special walls have been discovered surrounding them.

In the Polypi, inarticulate, fleshy bodies, having a simple visceral cavity, with a single opening at the centre above, without intestines, without glands separate from the walls of the visceral cavity, with no distinction of sex, and an imperfectly developed nervous system in the highest, and none whatever in the lowest, the circulatory system is rudimentary, and the fluid which it distributes nothing but the digested matters of the visceral cavity. The tubular axis of the collonial polyps communicates with the visceral cavities of all the individuals which compose the colony, and an oscillatory movement of the products of digestion which it contains, without any elaboration by special organs, and without any special organs of circulation, reminds us strongly of the motion of the fluid contents of the cells in the vegetable kingdom. In most of the invertebrata the digestive sac is surrounded by a cavity or sac called the visceral cavity. In many polyps these two cavities communicate freely. In the higher invertebrate animals these two cavities are separated from each other, and the nutritive materials pass into the exterior visceral cavity only by transudation through the walls of the stomach and intestinal tube. In certain Crustacea and the lowest Mollusca, the movement of these nutritive matters backwards and forwards, by the irregular contractions of their bodies, constitutes the only means by which their tissues are supplied with nutriment, and even in the higher Mollusca, Insects and Crustacea, the circulatory system is in free communication with the visceral cavity. In all these animals there is no other absorption but that which takes place through the walls of the alimentary canal into the digestive cavity.

Even in the Echinodermata and Annelida, which have a closed circulatory system, the principal channel for the transmission of the nutritive materials through the system appears to be from the visceral cavity. In these animals, however, absorption must also take place, to some extent, from the blood vessels, minutely distributed over the intestinal tube.

From this hasty review of our knowledge of the invertebrate animals, we see that their circulatory apparatus, organs and tissues, are not sufficiently developed and perfected, to call for the existence of a lymphatic system.

One office of the lymphatics is the absorption of fatty matters after they have formed an emulsion with the pancreatic fluid. In most invertebrate animals, the conditions for the formation of this emulsion and its absorption do not exist, because the digested matters pass directly from the alimentary canal and visceral cavity, into the blood-vessel system, without absorption.

In the four great classes of vertebrate animals the circulatory system is completely separated from the digestive cavity, all the organs and apparatuses are highly developed, and the existence of a special system of absorbents appears to be absolutely necessary for the preservation of the integrity of the fluids of the animal economy, and also for the absorption of fatty matters, which are of great importance in the maintenance of animal temperature.

In Fishes, the development and perfection of the pancreas correspond in no degree with the position occupied by different individuals in the classification of naturalists. The most superficial examination of the gland under consideration, will show that many animals of an exceedingly simple construction often have individual organs more highly developed, than animals which stand far above them in physical and mental constitution.

Thus, in the lowest orders of the cartilaginous fishes—the Cyclostomi and Plagiostomi—it resembles in all respects this gland in the most highly organized mammalia. In the Sturiones its structure is somewhat simplified, and in the majority of osseous fishes it is reduced to its rudimentary form, consisting of cæca varying in number in different species, and opening into the duodenum, below the circular valve of the stomach; whilst in others, as observed by Cuvier in the conger eel, pike and carp, and by Muller, in the Ophisurus serpens, it is intimately associated with the intestinal mucous membrane, consisting of simple follicular depressions, lined with the peculiar cells, constituted to secrete the pancreatic fluid. It cannot, therefore, be asserted as a universal law without any exception, that there is a regular progression in the development of the different organs in animals corresponding to the position which they occupy in the scale of creation. It matters not whether we view the pancreas in its earlier stages of development in the higher animals, or in its permanent condition in many of the osseous fishes, its structure is the same.

By classifying this organ according to its development in fishes, we have an exact history of the changes which occur during the development of this gland in the higher animals. The permanent forms of the pancreas of the former are but transitory conditions, forming the stages in the development of this organ in the latter. According to Muller, Weber and Wharton Jones, in higher animals, which have a perfect pancreas, its development, like that of the salivary glands, commences by a simple diverticulum, or cæcum, from the walls of the duodenum. This subdivides into bud-like processes. As the development of the gland advances, the canal and its branches become more and more ramified and subdivided, until the compound racemose lobulated gland is formed. Precisely the same stages of development in a permanent form, are discernible in the different orders, genera and species of Fishes.

Mere cells or follicular depressions in the mucous membrane of the small intestines, according to the observations of Cuvier, Muller and Solly, perform the offices of the pancreas in several species, as the Hyppoglossus rondeletus, conger eel, pike and carp, and Ophisurus serpens.

In the Ammodytes tobianus there is a single cæcum prolonged into a pouch, representing this gland in its rudimentary condition.

In the Octopus the single pancreatic appendage is prolonged and specially convoluted. Five of these cæca occur in Salmo spirinchus, six in Perca lucioperca and Sargus annularis, seven to eight in the bass, (Corvina ocellata,) and ten to thirty and more in many salmons and herrings, and from eighty to ninety in the common salmon.

Several species of Plaice have two, whilst others have three, like the river perch and common loach, whilst the Platessa oblongata has four of these cæcal pouches, which open into the pylorus and duodenum.

In Gadus and Scomber the number is greatly augmented, and the complexity of the gland increased by the division of the cæca. In Scomber thynnus four large trunks arise from the intestine and divide into branches, each of which subdivide and terminate at length in a tuft-like fasciculous of narrow tubular cæca.

In the salt-water garfish, (Lepisosteus osseus) of Georgia, the pancreas is situated with its superior convex border in contact with the inferior concave border of the liver, which resembles in shape and appearance this organ in serpents. The inferior border is in contact with the spleen. Upon the exterior it consists of numerous short cæca, which radiating inwards, unite together forming several branches, which again unite and constitute one short duct, having a diameter almost equal to that of the small intestine into which it opens. The duct branches, and cæca generally contain, especially after a meal of fish, a cream-like fluid, which, under the microscope, is found to be a true emulsion, containing innumerable minute globules of oil in a transparent fluid. The large opening of the duct of the pancreas is so situated just at the bend of the duodenum, that all the digested food, after passing the stomach, must be submitted to the influence of its secretion, and much passes into the duct and cæca. All the oleaginous matters must therefore be brought into contact with the pancreatic juice, and in this manner an emulsion is formed and prepared for absorption. The emulsion is not found in the stomach above the opening of the duct of the pancreas, but exists in greatest abundance in its immediate vicinity and within the cæca.

The structure of this gland in the fresh-water Garfish of the swamps of Georgia, is constructed on a similar plan; its cæca, however, are longer, and the branches more distinct. The duct branches and cæca contained in every instance, after a full meal, a similar fatty emulsion.

In the sword-fish (Ziphias gladius), this organ is very large, and Professor Grant states, in his lectures upon comparative anatomy, that it weighs six ounces more than the liver. It consists of innumerable small cæca, connected together by cellular tissue, in which ramify the capillary vessels. These cæca form a reniform mass, which is surrounded with a muscular tunic and the peritoneum. When opened, the innumerable component cæca are found to be formed by the successive divisions of the single great duct, into which they all pour their secretions into the duodenum immediately below the pyloric valve.

In the sturgeon, the structure of the pancreas is similar to that of the Ziphias gladius. The hundreds of cæca ramified from one common duct, are enclosed in a muscular and peritoneal coat. The contraction of the muscular tunic compresses the cæca and forces their secretion into the intestinal canal. The excretory duct opens close to the pyloric valve, and the termination of the ductus communis choledochus.

In the eel, pike, and fresh-water trout, we find a yellowish white compact and glandular pancreas, having from two to three excretory ducts, which are frequently accompanied in their course to the intestine by the biliary ducts. In the trout and some others there exists both pyloric appendages and a compact pancreatic gland. In the hammerhead shark (Zygæna malleus), we find an elongated, narrow, flattened, light yellow, compact pancreas, with little lobulation.

In the stingray, this organ is of a yellow color, well defined, lobulated form, resembling in all respects the perfectly developed pancreas of the Mammalia and other vertebrata.

The pancreas of the doubtful reptiles assume the appearance presented by that of the sturgeon, shark, and warm-blooded animals.

In the Menobranchus maculatus it is an irregularly shaped gland, having four principle lobes diverging from each other at right angles, thus presenting a stellated arrangement. The pancreas of the hellbender, (Menopoma alleganensis,) is a long delicate light yellow gland, which commences near the pyloric extremity of the stomach, and extends down along the duodenum and small intestine for about three inches. Its inferior portion is more expanded than the superior. The pancreas of the congo snake (Amphiuma means), is similar in structure and appearance to that of the Menopoma alleganensis, with the exception that it is broader and thicker.

In the Batrachia, the pancreas present a developed appearance, and generally commences by a small slender lobe, at the pyloric extremity of the stomach, and passing downwards and forwards expands into a broad lobulated mass. As in many other cold-blooded animals, it is not in contact with the spleen.

In all the American Ophidia which I have had the opportunity of examining, as the banded rattlesnake (Crotalus durissus), the water rattlesnake (Crotalus adamanteus), ground rattlesnake (Crotalophorous miliarius), water mokeson (Trigonocephalus piscivorous), copperhead (Trigonocephalus contortrix), hognose viper (Heterodon platyrhinus,) black viper (Heterodon niger), grass snake (Tropidonotus ordinatus), (Tropidonotus fasciatus), green snake (Leptophis æstivus), coach-whip snake (Psammophis flagelliformis), indigo snake (Coluber Couperi), chicken snake (Coluber quadrivittatus), corn snake (Coluber guttatus), black snake (Coluber constrictor), the pancreas is a compact ovoid gland, often kidney-shaped, situated in contact superiorly with the gall bladder, and inferiorly attached to the duodenum. The spleen, which is very small in these animals, is attached to the antero-superior surface of the pancreas. The hepatic and cystic ducts perforate the substance of the pancreas, and, uniting with its duct, enter the duodenum.

In the carnivorous Chelonia, the pancreas is a large, well developed, light yellow lobulated gland.

In the soft-shelled terrapin (Trinonyx ferox), it commences opposite the pyloric valve of the stomach. The principal lobe extends down along the small intestine about three inches. At the inferior portion it sends off two lobes. The inferior one short and broad; the superior longer, and passing downwards comes in contact with the spleen and passes along the anterior surface of this organ. The structure, position and appearance of this gland does not differ in any essential respect in the Alligator cooter, (Chelonura serpentina,) loggerhead turtle (Chelonia caretta), salt water terrapin (Emys terrapin), chicken terrapin (Emys reticulata), yellow belly terrapin (Emys serrata), and other carnivorous terrapins. In the gopher, however, which is the only herbivorous Chelonian in Georgia, the size and appearance of the pancreas is far different. It is a long, delicate gland, consisting of several slender and thin lobes, sub-divided into numerous small lobules.

Its size is far smaller than that of carnivorous terrapins. The reason of this will be readily understood when we consider the functions of the gland.

In birds the pancreas is a conglomerated gland, generally of large size, and is invariably lodged within a loop formed by the duodenum. It generally consists of two portions or lobes, united by a slender isthmoid partition. In some individuals it is single, and in others consists of three lobes. From each lobe an excretory duct is given off. These ducts terminate separately in the intestine near the opening of the biliary canals.

The color and appearance are similar to that of the well developed pancreas in all animals, cold or warm-blooded, and it is remarkable how constant is the color of the different glands in vertebrate animals. Any one familiar with comparative anatomy and physiology, will distinguish the different organs in any animals at a single glance. How uniform must be the operations of nature, when even the color of organs are definite and immutable, and have remained thus from the creation.

The pancreas of the omnivorous and carnivorous Mammalia, resembles in appearance and structure that of man, and its secretion enters the duodenum at the same point as that of the liver. In the apes, the Ruminantia, and most Carnivora and Rodentia, it has but one duct, which usually unites with the biliary. In some individuals, as the horse, hog, otter and beaver, it has two ducts, one of which unites with the biliary duct, and the other enters by itself, farther behind into the duodenum. In the rabbit, the biliary and pancreatic ducts are separated from each other by a considerable interval.

The pancreas of all the carnivorous Mammalia, which I have thus far examined, is much larger than that of the frugivorous Mammalia. This illustrates an important physiological fact, and will be demonstrated by numerous comparative weights of the organ, accurately ascertained.

Having considered the developement, structure and comparative anatomy of the pancreas in the four great classes of vertebrate animals, we will next study its use in the animal economy. Although Mayer, Magendie, Tiedeman, Gmelin, Leuret, Lassaigne, and other physiologists and chemists had investigated the physical and chemical properties of the pancreatic fluid, still one of its important offices was entirely unknown, until the researches of M. Cl. Bernard,* demonstrated that it is indispensable for the formation of chyle and the absorption of fatty matters. Previous to this discovery, it was considered similar to the fluid secreted by the salivary glands, and its principal use was affirmed to be the conversion of starch into glucose.

The investigations of M. Cl. Bernard, demonstrated that the limpid chyle (formerly called vegetable chyle), is the product of the digestion of materials which contain no fatty matters, and the white chyle (called formerly animal chyle), contains fatty matters in the state of an emulsion, and the lymphatics of the mesentery are found to contain a white milky fluid, only after the absorption of fatty matters, and that this emulsion and modification of the fatty matters were effected by the agency of the pancreatic juice. These conclusions were derived from the results of numerous ingenious experiments.

If dogs are fed upon oleaginous matters and killed at different periods, oil will be found unaltered until it comes in contact with the pancreatic fluid, and if the pancreatic ducts be tied, all alteration is prevented and the oil remains transparent.

The most conclusive and beautiful of all Dr. Bernard's experiments were performed upon the rabbit.

In this animal, the pancreatic duct opens into the intestine very low down, from six to fourteen inches below the hepatic duct; and if fatty matters be introduced into the stomach, and the animal killed in three or four hours, they will be found to have become emulsioned, and the lymphatics of the mesentery filled with white chyle. M. Cl. Bernard further showed, that if fatty bodies be exposed to the pancreatic fluid out of the body, a complete emulsion is formed; and if it be allowed to remain long enough, the fatty substances will be decomposed into glycerine

*Annales des Sciences Natur., Sept., 1848.

and fatty acids, and in the case of butter, butyric acid. Parallel experiments instituted with other fluids, as bile, saliva, gastric juice, serum of the blood, produce no such effects on fatty bodies. It is probable that M. Bernard supposed that fats were in the animal economy resolved into glycerine and fat acids. This process, however, would be very complicated, and involves many difficulties, and it is more reasonable to suppose that the action of the pancreatic juice is limited to the formation of an emulsion, which is nothing more than the mechanical division of the fat into minute globules, coated with a thin film of the albuminoid elements of the pancreatic juice. That this is really the case in living animals, I have enjoyed many opportunities of demonstrating whilst examining the pancreas of the garfish, (Lepisosteus osseus.) In this fish, the duct of the pancreas has a diameter almost equal to that of the intestine, and is so situated that all the digested matters which pass out of the stomach must come in contact with its secretion, and often pass in considerable amount into the duct and cæca of the gland. When the emulsion is squeezed out of the duct and cæca, and subjected to the microscope, it is found to consist of innumerable minute globules of oil, surrounded by a transparent fluid.

The correctness of M. Bernard's observations have been called in question by Dr. Bence Jones, Lenz, Frerichs, Bidder, Schmidt, Lehmann, Donders and Herbert. It is asserted that the bile and intestinal juice are even more active and efficient than the pancreatic juice in the preparation of fatty matters for absorption.

It is objected to Bernard's experiments, that he delayed his examination of the animals too long, and allowed the emulsion formed with the bile to pass down and be absorbed before inspecting the viscera of the rabbits. Dr. Samuel Jackson,* Professor of the institutes of Medicine in the Univerity of Pennsyl vania, has recently examined this subject carefully, and repeated the experiments of Bernard, avoiding every source of error, and especially that of time, by causing oleaginous matters to enter the digestive apparatus constantly, until the moment of observation. In every instance, the results of his experiments confirmed the correctness of Bernard's conclusion that the emul-

* American Journal of Medical Sciences, October, 1854, page 307.

sion of fatty matters is produced by the action of the pancreatic juice.

After exposing the errors of the experiments upon which the German physiologists found their opposition to this doctrine, the Doctor concludes his valuable paper by the following summary of the present state of our knowledge :—

1. "Liquid fats are not miscible with the aqueous albumino-saline fluid—liquor sanguinis—with which all the vascular tissues are saturated; it cannot enter their pores, and consequently cannot be absorbed.

2. Liquid fats, when emulsified by albumen, are reduced to minute particles, each coated with albumen. In this state they are miscible, with the liquor sanguinis moistening the tissues, can enter their pores, and are then capable of absorption. This is the sole condition requisite for the absorption of fats."

3. "The white, milk-like fluid named chyle is this emulsion of the fatty matters of the food, mixed with the ordinary lymph, always contained in the lymphatics of the alimentary canal and other abdominal organs and mesentery. The molecular base of Gully is the microscopic appearance in the chyle of the minute globules of fat coated with albumen.

4. "Albumen forms a perfect and persistent emulsion with oils. The pancreatic fluid is a saturated albuminous solution, and forms with oils an emulsion equally as perfect and permanent as that of albumen.

5. "The pancreatic juice is the only highly albuminous fluid in the alimentary canal, and can accomplish the formation of a perfect emulsion; and the opinion of M. Cl. Bernard, that this process is one of its functions, is, it appears to me, sustained.

6. "The observations of M. Cl. Bernard, that the formation of the emulsion of fats in rabbits, is at and below the pancreatic duct, and not above it, is confirmed by the experiments reported in this communication. And, further, that the experiments upon rabbits are the most reliable, as being a true exemplification of the natural process, unattended with violence and torture to the animals, more or less disturbing in their effects.

7. "That M. Cl. Bernard's view of the decomposition of fats by the pancreatic juice is not proved, is opposed by the nature of the process and by analogy with other emulsions; it is unnecessary to the accomplishment of the absorption of fats, and introduces other and complicated processes that are unknown to exist, and are mere hypotheses."

Whilst engaged last summer in the investigation of the physical and chemical constitution of the fluids, and the comparative anatomy and physiology of cold-blooded animals, it occurred to me that the pancreas of cold-blooded animals should be larger than that of the frugivorous or granivorous animals, because it is much more incessantly exercised in the secretion of a fluid for the emulsifying of fats. Accordingly, I ascertained accurately the weights of the body and pancreas of every animal that fell into my possession. Dividing the weight of the former by that of the latter, we obtain the weight of the pancreas in relation to that of the body, and the relative size of this organ in different animals is thus ascertained. The weights of the animals were taken upon a pair of scales capable of turning to half a grain, and the weights of the organs were taken upon a delicate balance, capable of turning to one-thousandth of a grain.

The following table contains the most important results thus obtained.

*Comparative weight of the Pancreas of Carnivorous Fishes and Reptiles.*

| | | Times the weight of its Pancreas. |
|---|---|---|
| Weight of | Female Stingray (Trygon sabina) | 1071. |
| " | Hammerhead Shark (Zygæna malleus) | 1045. |
| " | Hammerhead Shark (Zygæna malleus) | 1563. |
| " | Female Garfish (Lepisosteus osseus) | 193. |
| " | Female Garfish (Lepisosteus osseus) | 212. |
| " | Bull Frog (Rana pipiens) | 1088. |
| " | Black Viper (Heterodon niger) | 537. |
| " | Coachwhip Snake (Psammophis flagelliformis) | 1353. |
| " | Corn Snake (Coluber guttatus) | 1371. |
| " | Black Snake (Colluber constrictor) | 472. |
| " | Banded Rattle Snake (Crotalus durissus) | 965. |
| " | Loggerhead Turtle (Chelonia caretta) | 518. |
| " | Alligator Cooter (Chelonura serpentina) | 630. |
| " | Salt-water Terrapin (Emys terrapin) | 994. |
| " | Chicken Terrapin (Emys recticulata) | 763. |
| " | Yellow-belly Terrapin (Emys serrata) | 1067. |
| " | Male Yellow-belly Terrapin (Emys serrata) | 1200. |
| " | Female Yellow-belly Terrapin (Emys serrata) | 1343. |

*Comparative weight of the Pancreas of Frugivorous Chelonians.*

| | Times the weight of its Pancreas. |
|---|---|
| Weight of Male Gopher (Testudo polyphemus) | 3500. |
| " Male Gopher (Testudo polyphemus) | 3061. |

*Comparative weight of the Pancreas of Carnivorous Mammalia.*

| | Times the weight of its Pancreas. |
|---|---|
| Weight of Female Raccoon (Procyon lotor) | 241. |
| " Female Raccoon (Procyon lotor) | 155. |
| " Fetus of Raccoon (Procyon lotor) | 583. |
| " Common Cat | 402. |
| " Pointer Dog | 337. |
| " Opossum (Didelphis virginianus) | 192. |

*Comparative weight of Pancreas of Frugivorous and Granivorous Mammalia.*

| | Times the weight of its Pancreas. |
|---|---|
| Weight of Common Sheep | 1125. |
| " Grey Squirrel (Sciurus carolinensis) | 3026. |

By comparing these numbers carefully, we are taught the following facts and conclusions:

1. The pancreas of the garfish, (Lepisosteus osseus,) a powerful, voracious and active fish, is much larger than that of more sluggish fishes. The garfish consumes large numbers of small fishes, which it readily captures with its long and well armed jaws. The tissues and organs, especially the liver of fishes, contain much oil, and consequently a large gland is needed to afford a sufficient amount of the peculiar matters absolutely requisite for the preparation of the oleaginous matters for absorption. We have previously stated that, in the pancreas of this remarkable fish, we have an absolute demonstration of the function of this gland.

2. The pancreas of carnivorous fishes and reptiles is relatively much larger than that of frugivorous Chelonians. This difference in the relative size of this organ in these two classes, is evident at a glance. In the Ophidians it is a compact ovoid gland, and in the carnivorous Chelonians it is a broad lobulated, well developed, conspicuous gland, whilst in the frugivorous Gopher (Testudo polyphemus) it is a thin, delicate, obscure gland, composed of several slender lobes, subdivided into numerous small lobules.

3. The pancreas of carnivorous mammalia is much larger than that of the frugivorous or granivorous. The strongest exception to this assertion appears to be in the beaver, which is stated to have an unusually large pancreatic gland. A consideration of the character of the food of this animal, will, we think, explain this anomaly. In this animal the stomach, and more especially the cæcum, is stated by observers to be plugged up with fragments of bark and wood, which appear to constitute its chief aliment. The experiments of Mitscherlich have shown that alkaline solutions are capable of converting cellulose into starch even more readily than concentrated acids. It is therefore highly probable that the great office of the alkaline pancreatic fluid in these animals, is the preparation of cellulose for absorption by converting it into starch.

4. The pancreas of carnivorous fishes and reptiles is larger than that of frugivorous and granivorous mammalia, notwithstanding that the *digestion* of the former is much slower than that of the latter, and the amount of nutritive matters necessary to sustain the economy much less.

5. The pancreas of carnivorous mammalia is larger than that of carnivorous, cold-blooded animals, because the digestive process is much more rapid, and correspondingly larger glands are needed to supply the secretions necessary for the proper preparation of the food for absorption.

The difference between the weights of the organ in these two classes of animals, does not correspond exactly with the disparity of their respective digestive processes, because the sluggish circulation and æration of the blood, and the small amount of nervous force possessed by cold-blooded animals, require much larger organs to accomplish precisely the same results. As circulation and respiration are developed and perfected, and all the acts of life rendered correspondingly active and energetic, the more perfect and condensed become the organs and apparatuses.

These results were demonstrated, as far as simple dissection and inspection could show them, upon other animals, killed in swamps and woods, and at periods when it was impossible to ascertain their weights.

Our investigations upon birds have not been sufficiently extended to warrant general conclusions.

Whilst experimenting upon the effects of starvation and a change of diet upon the blood of carnivorous terrapins, I found that the pancreas of many of those which had been deprived of food and drink for a length of time, and then placed in a tub of water and liberally supplied with vegetable food, (purslain, Portulacca oleracea,) was diseased. Parts of the gland were of a black color and hard texture, and under the microscope contained cancer cells and crystals, which resembled in appearance those of the triple phosphate. It is probable that the gland, not being normally exercised, degenerated in structure.

In the first of a series of experiments which have not as yet been completed, I ascertained the correctness of M. Cl. Bernard's statement, that fatty matters are not altered in the stomach or intestines of dogs, if the pancreatic duct be tied.

The abdominal cavity of a remarkable large and voracious pointer dog, noted for his powerful digestive powers was opened along the linea alba, and f.℥ii of lard oil secured in the stomach, by ligatures above and below, and f.℥i were injected and secured in the same manner, in the intestines. The viscera were then carefully returned and the wound sowed up. At the expiration of six hours, the dog was killed, and the contents of the stomach and intestines had neither increased nor diminished, and were changed neither in physical or chemical properties, and the lymphatics of the mesentery did not appear to contain any milky emulsion. Under the microscope, the lard oil presented an appearance differing in no respect from that of ordinary oil. Lard oil was enclosed separately in the stomach and intestines of a dog, and immersed for eighteen hours in the serum of this animal. At the end of this time neither endosmose of the serum, nor exosmose of the oil, had taken place. In the living dog, the blood-vessels of the stomach and intestines retained their natural size and appearance. When saline solutions of high specific gravity were enclosed in a similar manner in the stomach and intestines of dogs and cats, the blood-vessels were congested with blood, and the internal surface of the mucous membrane presented a pinkish purple color.

The following general conclusions have been drawn from this study of the comparative anatomy and physiology of the pancreas.

1. In the invertebrate animals, this gland and the lymphatic system do not exist, because the character of the circulatory system and the manner in which it receives the digested matters from the visceral cavity, are such, that the conditions requiring their presence do not exist.

2. In fishes we may study the development of the pancreas, the permanent forms being but the transient conditions in the development of this gland in the higher animals.

3. The assertion of M. Cl. Bernard, that the chief office of the pancreas, is to prepare fatty matters for absorption is sustained by the following facts.

*a.* In the garfish (Lepisosteus osseus,) the emulsion of the fatty matters takes place in the duct and cæca of the pancreas, and their immediate vicinity, and nowhere else in the alimentary canal.

*b.* The pancreas of carnivorous animals is relatively much larger than that of frugivorous and granivorous animals. The amount of oil consumed by the former is much greater than that consumed by the latter. It is reasonable to infer from these facts, that the principal office of the pancreatic juice, is the preparation of fats for absorption. This is farther sustained by the fact that the size of the pancreas amongst carnivorous animals is in a measure proportional to the amount of oleaginous matters consumed. The pancreas of the active, voracious gar-fish who destroys large numbers of small fish, is larger than that of the more sluggish fishes.

*c.* The pancreas of carnivorous Chelonians, fed upon vegetable matters, degenerated in its structure.

